# Theoretical analysis of a circular hybrid plasmonic waveguide to design a hybrid plasmonic nano-antenna

**DOI:** 10.1038/s41598-020-71863-5

**Published:** 2020-09-15

**Authors:** Maryam Khodadadi, Najmeh Nozhat, Seyyed Mohammad Mehdi Moshiri

**Affiliations:** Department of Electrical Engineering, Shiraz University of Technology, 7155713876 Shiraz, Iran

**Keywords:** Nanophotonics and plasmonics, Nanophotonics and plasmonics

## Abstract

In this paper, a circular hybrid plasmonic waveguide-fed nano-antenna (CHPWFNA) has been introduced for operating at the standard telecommunication wavelength of 1,550 nm. For the first time, the dispersion relation of a circular hybrid plasmonic waveguide as the feed line of the proposed nano-antenna has been derived, analytically. To verify the accuracy of the analytical solution, two numerical techniques of finite element method (FEM) and finite-difference time*-*domain (FDTD) method have been used. Numerical results are well-matched with the theoretical ones. The characteristics of the CHPWFNA have been studied by two mentioned methods. The obtained realized gains (directivities) by the FDTD and FEM simulations are 9.03 dB (9.38 dBi) and 10.00 dB (10.32 dBi), respectively, at 1,550 nm wavelength. For on-chip point-to-point wireless link performance, the obtained quality factor by the FDTD method (FEM) is 63.97 (100). The obtained radiation characteristics and link performance reveal that at 1,550 nm, the proposed antenna has the best performance. Besides, the frequency bandwidth of the antenna (185–200 THz) covers the low-loss optical frequency range. Also, paying attention to the laser eye safety is so important. Consequently, the wavelength of 1,550 nm has been chosen as the target wavelength. Moreover, the array configuration has been studied and the directivity and realized gain have been obtained based on the array factor theory and numerical methods, which are agree with each other. The attained realized gain by the FDTD method (FEM) for the considered single row array, at 1,550 nm, is 11.20 dB (11.30 dB). There is a little difference between the numerical results due to the total mesh size, the grid size refinement and the relative error of the numerical methods convergence. Finally, as one of the most important challenges in fabrication is the gold surface quality, we have studied the effect of gold surface roughness and its pentagonal cross section on the antenna performance.

## Introduction

Surface plasmon polaritons (SPPs) have extensively attracted scientists’ interest over the years because they opened a new promising approach for next generation of nanotechnologies. The integration and miniaturization of photonic components and circuits are an integral part of the science development. Therefore, utilizing SPPs guarantee the requirements of nanophotonics branch^1^ to design efficient nano devices^2^ with ultra-fast operational speed^3^, mechanical flexibility^4^, low losses^4^ and the ability to confine the light into a region much smaller than the operating wavelength^5^. As a result, SPPs remarkably have been utilized in a variety of applications such as nano-lasers^6^, nano-antennas^7^, waveguides^8^ and modulators^9^.

Plasmonic waveguides (PWs) and waveguide-fed nano-antennas^10^ support highly localized optical fields at the interface of metal-dielectric layer in the visible and infrared (IR) regions^11^. However, the light propagation length of PWs is very short in comparison to the optical fibers and dielectric waveguides (DWs) due to the metallic attenuation^12,13^. Basically, the benefits and restrictions of PWs and DWs are in some ways supplementary^14^. On one hand, although DWs are lossless, their mode size is constrained by the diffraction limit. On the other hand, PWs can concentrate the light beyond the diffraction limit but they suffer from the high propagation loss. Therefore, in order to have a better compromise between the inevitable metallic loss and fundamental mode confinement in a nano-scale layer, in comparison to PWs, hybrid plasmonic waveguides (HPWs) have been proposed and demonstrated^15^. As an outstanding endeavor, HPW has been designed by combining the PW and DW. The utilized DW in HPW consists of a dielectric slab surrounded by the medium with low refractive index, which can support transverse magnetic (TM) mode. When two waveguides (PW and DW) are brought close to each other, the SP mode supported by the PW is coupled with the TM mode of the DW and forms a hybrid mode^14^ at the midpoint of the low refractive index layer (coupling region). Fundamentally, previous studies have shown that HPWs can support a hybrid plasmonic quasi-transverse magnetic mode, which is confined in a low-index dielectric layer sandwiched between the noble metal and high-index dielectric layers^15–19^ because the SP mode is TM in nature. As a result, the advantages of both DWs (lossless feature) and PWs (overcoming the diffraction limit) have been combined by introducing the HPWs^15^.

Also, the propagated hybrid mode consists of two even and odd coupled modes for similar PW and DW^14^. Although, for dissimilar waveguides in the coupling region, the hybrid coupled mode does not have complete symmetry, which consists of the “quasi-odd” (becomes zero somewhere in the coupling region) and “quasi-even” (does not become zero anywhere in the coupling region) modes^14^. As an important point, it should be mentioned that HPWs support the quasi-even mode for any thickness of the coupling region compared to the quasi-odd mode, which does not exist for small coupling region thickness^14,15^. Up to now several HPWs have been introduced to increase the propagation length of SPPs and confine the light in the coupling region^14–19^. For example, rectangular silicon- or InP-based waveguides covered by metal cap^16,17^, a metal wire covered by low- and high-index dielectric layers^18^ and a high-index dielectric cylinder covered by low-index dielectric and noble metal layers^19^ are famous structures of HPWs.

Recently, hybrid plasmonic waveguide-fed nano-antennas have obtained significant attention due to their ability to convert free-propagating optical radiation into localized energy and vice versa through the reciprocity theory, in addition to focusing on controlling the distribution of the local field^20–26^. Various topologies for hybrid plasmonic nano-antennas (HPNAs) have been proposed based on the design of rectangular hybrid plasmonic waveguide-fed (RHPW) antennas such as patch^20,22^, Vivaldi^23^, bowtie^21^, horn-like^24^, V- and W-shaped^25,26^. To design HPNAs for optical wireless communications, the critical parameters of gain, directivity, efficiency, propagation length and impedance matching play important roles.

A silicon-based RHPW patch nano-antenna and a multilayer patch nano-antenna have been proposed at 193.5 THz with a narrow frequency bandwidth and gains of 5.65 and 8.30 dB, respectively^20,22^. A wideband HPNA, which covers all optical communication frequencies with the gains of 2.06, 4.64 and 5.03 dB at 193.5, 229 and 352.9 THz, respectively, have been introduced^23^. Nevertheless, in comparison to refs.^20,22^, the gains have not been increased remarkably at the frequency of 193.5 THz. Based on the introduced RHPW in ref.^24^, a wideband HPNA in the frequency range of 160 to 400 THz has been studied with the gains of 4.67, 7.26 and 4.80 dB at 193.5, 229 and 352.9 THz, respectively, with smaller foot-print. A new approach has been opened by introducing an InP-based RHPW nano-antenna^21^. The proposed bowtie HPNA is capable of monolithic integration with laser and photodetector at 193.5 THz with the gain of about 9 dB in the frequency range of 180 to 220 THz^21^. In our previous work, we have introduced a new vertical director to increase the directivity of the InP-based W-shaped nano-antenna to the values of 7.96 and 10.72 dBi at the wavelengths of 1,310 and 1,550 nm, respectively^25^. Moreover, based on the coupled HPW, a V-shaped nano-antenna has been proposed, which is able to receive/transmit optical signals at three telecommunication wavelengths of 850, 1,310, and 1,550 nm with the high realized gains of 10.50, 9.39, 9.05 dB, respectively, the maximum efficiency of 98%, and ∼86% bandwidth from 150 to 390 THz^26^.

In this paper, a circular hybrid plasmonic waveguide-fed nano-antenna (CHPWFNA) has been proposed and analyzed. For the first time, the dispersion relation of the fundamental TM_01_ mode of the circular hybrid plasmonic waveguide (CHPW) has been derived analytically. The CHPW consists of a metal wire covered by low- and high-index dielectric layers. This design is an improved structure of a bare metal wire waveguide with a hybrid mode and a thin gold layer around a reduced cladding fiber. Moreover, to confirm the accuracy of our analytical method, we have calculated the dispersion relation of TM_01_ mode by the finite element method (FEM) and finite-difference time-domain *(*FDTD) method. The proposed CHPWFNA can radiate lightwave into free space in the frequency range of 185 to 200 THz with the maximum realized gains and total efficiencies of 9.03 dB (10.00 dB) and 92.25% (92.89%) for the FDTD method (FEM) at 193.5 THz, respectively. Also, the on-chip performance of the proposed nano-antenna has been investigated numerically and theoretically. Moreover, the applications of the proposed CHPWFNA for energy harvesting and beam-steering have been studied based on the array factor theory and mentioned numerical methods. Furthermore, the tolerance effect of geometrical parameters and the fabrication process have been demonstrated. Finally, the effects of gold surface quality and pentagonal cross section of the gold nanowire in the fabrication process as the crucial issues on the radiation characteristics have been studied.

## Theory of dispersion relation of circular hybrid plasmonic waveguide

**Figure 1:**
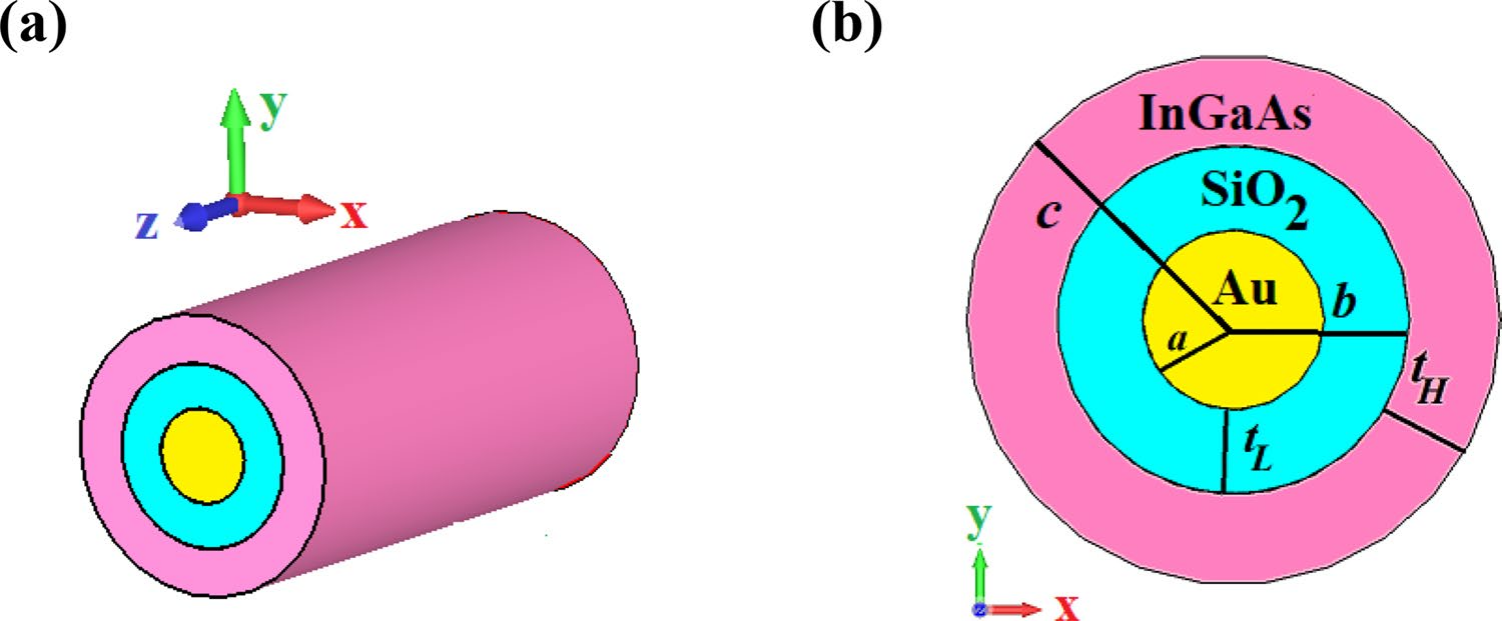
(**a**) The three-dimensional (3D) schematic view and (**b**) cross-section view of the proposed CHPW. The radius of gold layer and the thicknesses of SiO_2_ and InGaAs layers are *a* = 80 nm, $t_{L} = 75\,\,{\text{nm}}$ and $t_{H} = 80\,\,{\text{nm}}$, respectively.

The three-dimensional (3D) scheme and cross-section view of the investigated CHPW is depicted in Fig. 1. It is composed of a metal core, which is surrounded by low- and high-index dielectric layers of SiO_2_ and InGaAs, respectively. The proposed CHPW enables the SPPs guiding and subwavelength confinement of mode energy in the low-index dielectric region like the RHPW. The radius of the gold nanowire is *a* = 80 nm. The thicknesses of the SiO_2_ and InGaAs layers are $t_{L} = 75{\text{ nm}}$ and $t_{H} = 80{\text{ nm}}$ and their relative permittivities are $\varepsilon_{L} = 2.1055$ and $\varepsilon_{H} = 13.84$, respectively^18^ that are nearly frequency-independent and almost constant in the frequency range of 180 to 200 THz^27, 28^. For the Au layer, the relative permittivity $\left( {\varepsilon_{Au} } \right)$ is extracted from the Johnson-Christy data^29^.

To simplify the theoretical procedure for calculating the dispersion equation of the CHPW, the radius of the InGaAs layer (high-index dielectric layer) and the length of the CHPW along the z direction are considered infinite. Based on the hybrid plasmonic waveguide theory, the excited SPPs are confined in the layer with low refractive index and the amplitude of the SPPs decays exponentially in the metal and high-index dielectric layers. Therefore, the outer dielectric layer with infinite radius does not have major effect on the dispersion equation.

The longitudinal components of electric $\left( {E_{{z_{i} }} } \right)$ and magnetic $\left( {H_{{z_{i} }} } \right)$ fields in each layer of the CHPW can be written as follows:

1 $$E_{{z_{i} }} \left( {r,\varphi ,z} \right) = E_{{z_{i} }} \left( {r,\varphi } \right)e^{ - j\gamma z} \, \quad i = 1,2,3$$

2 $$H_{{z_{i} }} \left( {r,\varphi ,z} \right) = H_{{z_{i} }} \left( {r,\varphi } \right)e^{ - j\gamma z} \, \quad i = 1,2,3$$

where $\gamma = k_{0} \left( {n_{eff} + jk_{eff} } \right)$ is the complex propagation constant, $k_{0} = \frac{2\pi }{{\lambda_{0} }}$ is the free space wave number, and $\lambda_{0}$ is the free space wavelength. Also, $n_{eff}$ and $k_{eff}$ are the real and imaginary parts of the effective refractive index, respectively. Moreover, *r* and $\varphi$ are axial distance and azimuth angle in the cylindrical coordinate, respectively.

These longitudinal components satisfy the Helmholtz’s equations for each layer:

3a $$\nabla_{T}^{2} E_{{z_{i} }} + \left( {k_{i}^{2} - \gamma^{2} } \right)E_{{z_{i} }} = 0\, \,\quad i = 1,2,3$$

3b $$\nabla_{T}^{2} H_{{z_{i} }} + \left( {k_{i}^{2} - \gamma^{2} } \right)H_{{z_{i} }} = 0\, \, \quad i = 1,2,3$$

where $k_{i} = k_{0} n_{i}$ and $n_{i} = \sqrt {\varepsilon_{i} }$ are the transverse wave number and refractive index of each layer, respectively. Considering the cylindrical coordinate system and using the separation of variables method, Eq. (3) can be rewritten as:

4a $$\frac{{d^{2} E_{{z_{i} }} }}{{dr^{2} }} + \frac{1}{r}\frac{{dE_{{z_{i} }} }}{dr} + \left( {n_{i}^{2} k_{0}^{2} - \gamma^{2} - \frac{{m^{2} }}{{r^{2} }}} \right)E_{{z_{i} }} = 0\,$$

4b $$\frac{{d^{2} H_{{z_{i} }} }}{{dr^{2} }} + \frac{1}{r}\frac{{dH_{{z_{i} }} }}{dr} + \left( {n_{i}^{2} k_{0}^{2} - \gamma^{2} - \frac{{m^{2} }}{{r^{2} }}} \right)H_{{z_{i} }} = 0\,$$

where *m* is an integer number and corresponds to the variation of the fields relative to $\varphi$.

In General, the condition for the excitation of SPP modes in every waveguide such as CHPW is $\frac{\gamma }{{k_{0} }} > n_{i}^{2}$ or $n_{i}^{2} k_{0}^{2} - \gamma^{2} < 0.$ Therefore, the solution of Eq. (4) is modified Bessel function. It is essential to point out due to singularities of modified Bessel functions of the first kind at infinity, $\left( {I_{m} \left( r \right){\text{ at }}r = \infty } \right)$ and the second kind at origin $\left( {K_{m} \left( r \right){\text{ at }}r = 0} \right)$^30,31^, the longitudinal electromagnetic fields for each region are defined as follows:

5a $$E_{{z_{1} }} = A_{1} I_{m} \left( {\frac{{U_{1} }}{a}r} \right)e^{jm\varphi } e^{ - j\gamma z} , \, \,\,\, \, 0 \le r \le a$$

5b $$H_{{z_{1} }} = A^{\prime}_{1} I_{m} \left( {\frac{{U_{1} }}{a}r} \right)e^{jm\varphi } e^{ - j\gamma z} , \, \,\,\,\, \, \,\, \, 0 \le r \le a$$

6a $$E_{{z_{2} }} = \left[ {A_{2} I_{m} \left( {\frac{{U_{2} }}{b}r} \right) + A^{\prime}_{2} K_{m} \left( {\frac{{U_{2} }}{b}r} \right)} \right]e^{jm\varphi } e^{ - j\gamma z} , \, a < r \le b$$

6b $$H_{{z_{2} }} = \left[ {B_{2} I_{m} \left( {\frac{{U_{2} }}{b}r} \right) + B^{\prime}_{2} K_{m} \left( {\frac{{U_{2} }}{b}r} \right)} \right]e^{jm\varphi } e^{ - j\gamma z} , \, a < r \le b$$

7a $$E_{{z_{3} }} = A_{3} K_{m} \left( {\frac{{U_{3} }}{b}r} \right)e^{jm\varphi } e^{ - j\gamma z} , \, \,\,\, \, r > b$$

7b $$H_{{z_{3} }} = A^{\prime}_{3} K_{m} \left( {\frac{{U_{3} }}{b}r} \right)e^{jm\varphi } e^{ - j\gamma z} , \, \,\,\, \, \, \, r > b$$

where $$U_{1}^{2} = a^{2} \left( {\gamma^{2} - n_{1}^{2} k_{0}^{2} } \right) \ge 0$$, $$U_{2}^{2} = b^{2} \left( {\gamma^{2} - n_{2}^{2} k_{0}^{2} } \right) \ge 0$$ and $$U_{3}^{2} = b^{2} \left( {\gamma^{2} - n_{3}^{2} k_{0}^{2} } \right) \ge 0$$ are arguments of the modified Bessel functions in Au, SiO_2_ and InGaAs layers, respectively. Also, $$A_{i}$$, $$A^{\prime}_{i}$$, $$B_{i}$$ and $$B^{\prime}_{i}$$$$\left( {i = 1,\,\,2,\,\,3} \right)$$ are the amplitude of the electric and magnetic fields.

A considerable simplification occurs when we have used boundary conditions. The optical field of the fundamental guided mode (TM_01_) should be decay to zero rapidly for $r > b$ to satisfy the condition of the CHPW to confine the TM_01_ mode in the layer with low refractive index (SiO_2_ layer). Therefore, the boundary condition between the InGaAs and air has been ignored because the received field is zero at $r = c$. As a result, we have assumed that the thickness of the InGaAs is infinite and we have written the boundary condition of Eq. (7) for $r > b$ instead of $b < r \le c$.

In the CHPW only TM_01_ mode can be concentrated in the low-index dielectric layer, which is sandwiched between the gold nanowire and high-index dielectric layer. Therefore, for simplification in calculating the dispersion equation, we have considered *m* = 0 and so $K_{0}^{^{\prime}} (r) = - K_{1} (r)$ and $I_{0}^{^{\prime}} (r) = I_{1} (r)$. Utilizing Maxwell’s equations, the transverse components in each layer can be derived from $E_{{z_{i} }}$ and $H_{{z_{i} }}$:

For the metal nanowire core $\left( {0 \le r \le a} \right)$

8a $$E_{r1} = \frac{ - j}{{\left( {{{U_{1} } \mathord{\left/ {\vphantom {{U_{1} } a}} \right. \kern-\nulldelimiterspace} a}} \right)}}\gamma A_{1} I_{1} \left( {\frac{{U_{1} }}{a}r} \right)e^{ - j\gamma z}$$

8b $$E_{\varphi 1} = \frac{{j\omega \mu_{0} }}{{\left( {{{U_{1} } \mathord{\left/ {\vphantom {{U_{1} } a}} \right. \kern-\nulldelimiterspace} a}} \right)}}A^{\prime}_{1} I_{1} \left( {\frac{{U_{1} }}{a}r} \right)e^{ - j\gamma z}$$

8c $$H_{r1} = \frac{ - j}{{\left( {{{U_{1} } \mathord{\left/ {\vphantom {{U_{1} } a}} \right. \kern-\nulldelimiterspace} a}} \right)}}\gamma A^{\prime}_{1} I_{1} \left( {\frac{{U_{1} }}{a}r} \right)e^{ - j\gamma z}$$

8d $$H_{\varphi 1} = \frac{{ - j\omega \varepsilon_{0} \varepsilon_{Au} }}{{\left( {{{U_{1} } \mathord{\left/ {\vphantom {{U_{1} } a}} \right. \kern-\nulldelimiterspace} a}} \right)}}A_{1} I_{1} \left( {\frac{{U_{1} }}{a}r} \right)e^{ - j\gamma z}$$

For the low-index dielectric layer $\left( {a < r \le b} \right)$

9a $$E_{r2} = \frac{ - j}{{\left( {{{U_{2} } \mathord{\left/ {\vphantom {{U_{2} } b}} \right. \kern-\nulldelimiterspace} b}} \right)}}\gamma \left[ {A_{2} I_{1} \left( {\frac{{U_{2} }}{b}r} \right) + A^{\prime}_{2} K_{1} \left( {\frac{{U_{2} }}{b}r} \right)} \right]e^{ - j\gamma z}$$

9b $$E_{\varphi 2} = \frac{{j\omega \mu_{0} }}{{\left( {{{U_{2} } \mathord{\left/ {\vphantom {{U_{2} } b}} \right. \kern-\nulldelimiterspace} b}} \right)}}\left[ {B_{2} I_{1} \left( {\frac{{U_{2} }}{b}r} \right) + B^{\prime}_{2} K_{1} \left( {\frac{{U_{2} }}{b}r} \right)} \right]e^{ - j\gamma z}$$

9c $$H_{r2} = \frac{ - j}{{\left( {{{U_{2} } \mathord{\left/ {\vphantom {{U_{2} } b}} \right. \kern-\nulldelimiterspace} b}} \right)}}\gamma \left[ {B_{2} I_{1} \left( {\frac{{U_{2} }}{b}r} \right) + B^{\prime}_{2} K_{1} \left( {\frac{{U_{2} }}{b}r} \right)} \right]e^{ - j\gamma z}$$

9d $$H_{\varphi 2} = \frac{{ - j\omega \varepsilon_{0} \varepsilon_{L} }}{{\left( {{{U_{2} } \mathord{\left/ {\vphantom {{U_{2} } b}} \right. \kern-\nulldelimiterspace} b}} \right)}}\left[ {A_{2} I_{1} \left( {\frac{{U_{2} }}{b}r} \right) + A^{\prime}_{2} K_{1} \left( {\frac{{U_{2} }}{b}r} \right)} \right]e^{ - j\gamma z}$$

For the high-index dielectric layer $\left( {r > b} \right)$

10a $$E_{r3} = \frac{ - j}{{\left( {{{U_{3} } \mathord{\left/ {\vphantom {{U_{3} } b}} \right. \kern-\nulldelimiterspace} b}} \right)}}\gamma A_{3} K_{1} \left( {\frac{{U_{3} }}{b}r} \right)e^{ - j\gamma z}$$

10b $$E_{\varphi 3} = \frac{{j\omega \mu_{0} }}{{\left( {{{U_{3} } \mathord{\left/ {\vphantom {{U_{3} } b}} \right. \kern-\nulldelimiterspace} b}} \right)}}A^{\prime}_{3} K_{1} \left( {\frac{{U_{3} }}{b}r} \right)e^{ - j\gamma z}$$

10c $$H_{r3} = \frac{ - j}{{\left( {{{U_{3} } \mathord{\left/ {\vphantom {{U_{3} } b}} \right. \kern-\nulldelimiterspace} b}} \right)}}\gamma A^{\prime}_{3} K_{1} \left( {\frac{{U_{3} }}{b}r} \right)e^{ - j\gamma z}$$

10d $$H_{\varphi 3} = \frac{{ - j\omega \varepsilon_{0} \varepsilon_{H} }}{{\left( {{{U_{3} } \mathord{\left/ {\vphantom {{U_{3} } b}} \right. \kern-\nulldelimiterspace} b}} \right)}}A_{3} K_{1} \left( {\frac{{U_{3} }}{b}r} \right)e^{ - j\gamma z}$$

where $\varepsilon_{0}$, $\mu_{0}$, and $\omega$ are the vacuum permittivity, vacuum permeability and angular frequency, respectively.

By applying boundary conditions at the interfaces of $r = a$ (Eq. (S1) of SI) and $r = b$ (Eq. (S2) of SI) for satisfying the continuity of the tangential components of electric and magnetic fields across the boundaries $\left( {E_{{\varphi_{i} }} = E_{{\varphi_{i + 1} }} } \right.$, $\,H_{{\varphi_{i} }} = H_{{\varphi_{i + 1} }}$, $E_{{z_{i} }} = E_{{z_{i + 1} }}$, and $\left. {H_{{z_{i} }} = H_{{z_{i + 1} }} \left( {i = 1,\,\,2} \right)} \right)$, a set of linear equations with unknown coefficients in the form of $\left[ M \right]\left[ C \right] = \left[ 0 \right]$ are obtained, where $\left[ M \right]$ and $\left[ C \right]$ are named as characteristic and coefficients matrices, respectively. To obtain a nontrivial solution, the determinant of $\left[ M \right]$ must be vanished, which leads to the dispersion relation. For this structure, $\left[ M \right]$ is an 8 × 8 matrix for the TM_01_ mode. To simplify the calculations, we have decreased the dimension of the $\left[ M \right]$ and $\left[ C \right]$ to 4 × 4 matrices by considering the relation between some coefficients based on the boundary conditions at the interfaces of $r = a$ and $r = b$(Eq. (S3) of SI):

11a $$A_{1} = \frac{{A_{2} I_{0} \left( {U_{2} \frac{a}{b}} \right) + A^{\prime}_{2} K_{0} \left( {U_{2} \frac{a}{b}} \right)}}{{I_{0} \left( {U_{1} } \right)}}$$

11b $$A^{\prime}_{1} = \frac{{B_{2} I_{0} \left( {U_{2} \frac{a}{b}} \right) + B^{\prime}_{2} K_{0} \left( {U_{2} \frac{a}{b}} \right)}}{{I_{0} \left( {U_{1} } \right)}}$$

11c $$A_{3} = \frac{{A_{2} I_{0} \left( {U_{2} } \right) + A^{\prime}_{2} K_{0} \left( {U_{2} } \right)}}{{K_{0} \left( {U_{3} } \right)}}$$

11d $$A^{\prime}_{3} = \frac{{B_{2} I_{0} \left( {U_{2} } \right) + B^{\prime}_{2} K_{0} \left( {U_{2} } \right)}}{{K_{0} \left( {U_{3} } \right)}}$$

After considerable algebraic calculations, the 4 × 4 matrix for the TM_01_ mode is obtained as follows (Eqs. (S4)- (S7) of SI):

12 $$\left[ M \right]\left[ C \right] = \left[ 0 \right] \to \left[ \begin{gathered} a_{11} \, a_{12 \, } 0 \, 0 \hfill \\ 0 \, 0 \, a_{23} \, a_{24} \hfill \\ a_{31} \, a_{32} \, 0 \, 0 \hfill \\ 0 \, 0 \, a_{43} \, a_{44} \hfill \\ \end{gathered} \right] \, \left[ \begin{gathered} B_{2} \hfill \\ B^{\prime}_{2} \hfill \\ A_{2} \hfill \\ A^{\prime}_{2} \hfill \\ \end{gathered} \right] = [0]$$

where

13a $$a_{11} = \frac{{U_{2} }}{{U_{1} }}\frac{a}{b}\frac{{I_{1} \left( {U_{1} } \right)}}{{I_{0} \left( {U_{1} } \right)}}I_{0} \left( {U_{2} \frac{a}{b}} \right) - I_{1} \left( {U_{2} \frac{a}{b}} \right)$$

13b $$a_{12} = \frac{{U_{2} }}{{U_{1} }}\frac{a}{b}\frac{{I_{1} \left( {U_{1} } \right)}}{{I_{0} \left( {U_{1} } \right)}}K_{0} \left( {U_{2} \frac{a}{b}} \right) - K_{1} \left( {U_{2} \frac{a}{b}} \right)$$

13c $$a_{23} = \frac{{U_{2} }}{{U_{1} }}\frac{a}{b}\frac{{\varepsilon_{Au} }}{{\varepsilon_{L} }}\frac{{I_{1} \left( {U_{1} } \right)}}{{I_{0} \left( {U_{1} } \right)}}I_{0} \left( {U_{2} \frac{a}{b}} \right) - I_{1} \left( {U_{2} \frac{a}{b}} \right)$$

13d $$a_{24} = \frac{{U_{2} }}{{U_{1} }}\frac{a}{b}\frac{{\varepsilon_{Au} }}{{\varepsilon_{L} }}\frac{{I_{1} \left( {U_{1} } \right)}}{{I_{0} \left( {U_{1} } \right)}}K_{0} \left( {U_{2} \frac{a}{b}} \right) - K_{1} \left( {U_{2} \frac{a}{b}} \right)$$

13e $$a_{31} = I_{1} \left( {U_{2} } \right) - \frac{{U_{2} }}{{U_{3} }}\frac{{K_{1} \left( {U_{3} } \right)}}{{K_{0} \left( {U_{3} } \right)}}I_{0} \left( {U_{2} } \right)$$

13f $$a_{32} = K_{1} \left( {U_{2} } \right) - \frac{{U_{2} }}{{U_{3} }}\frac{{K_{1} \left( {U_{3} } \right)}}{{K_{0} \left( {U_{3} } \right)}}K_{0} \left( {U_{2} } \right)$$

13g $$a_{43} = \frac{{U_{3} }}{{U_{2} }}\frac{{\varepsilon_{L} }}{{\varepsilon_{H} }}I_{1} \left( {U_{2} } \right) - \frac{{K_{1} \left( {U_{3} } \right)}}{{K_{0} \left( {U_{3} } \right)}}I_{0} \left( {U_{2} } \right)$$

13h $$a_{44} = \frac{{U_{3} }}{{U_{2} }}\frac{{\varepsilon_{L} }}{{\varepsilon_{H} }}K_{1} \left( {U_{2} } \right) - \frac{{K_{1} \left( {U_{3} } \right)}}{{K_{0} \left( {U_{3} } \right)}}K_{0} \left( {U_{2} } \right)$$

Finally, the dispersion equation can be attained as:

14 $$\det (M) = a_{23} a_{44} - a_{11} a_{32} + a_{12} a_{31} - a_{43} a_{24} = 0$$

Based on the boundary conditions, if we assume that the values of $A^{\prime}_{3}$ and $A_{1}$ are one, other coefficients are obtained as follows:

15a $$A^{\prime}_{1} = \frac{{B_{2} I_{0} \left( {U_{2} \frac{a}{b}} \right) + B^{\prime}_{2} K_{0} \left( {U_{2} \frac{a}{b}} \right)}}{{I_{0} \left( {U_{1} } \right)}}$$

15b $$A_{2} = \frac{{I_{0} \left( {U_{1} } \right)}}{{I_{0} \left( {U_{2} \frac{a}{b}} \right) - \frac{{a_{23} }}{{a_{24} }}K_{0} \left( {U_{2} \frac{a}{b}} \right)}}$$

15c $$A^{\prime}_{2} = - \frac{{a_{23} }}{{a_{24} }}\frac{{I_{0} \left( {U_{1} } \right)}}{{I_{0} \left( {U_{2} \frac{a}{b}} \right) - \frac{{a_{23} }}{{a_{24} }}K_{0} \left( {U_{2} \frac{a}{b}} \right)}}$$

15d $$B_{2} = \frac{{ - a_{32} }}{{a_{31} }}\frac{{K_{0} \left( {U_{3} } \right)}}{{K_{0} \left( {U_{2} } \right)}}\left[ {1 + \frac{{a_{32} }}{{a_{31} }}\frac{{I_{0} \left( {U_{2} } \right)}}{{K_{0} \left( {U_{3} } \right)}}} \right]$$

15e $$B^{\prime}_{2} = \frac{{K_{0} \left( {U_{3} } \right)}}{{K_{0} \left( {U_{2} } \right)}}\left[ {1 + \frac{{a_{32} }}{{a_{31} }}\frac{{I_{0} \left( {U_{2} } \right)}}{{K_{0} \left( {U_{3} } \right)}}} \right]$$

15f $$A_{3} = \frac{{A_{2} I_{0} \left( {U_{2} } \right) + A^{\prime}_{2} K_{0} \left( {U_{2} } \right)}}{{K_{0} \left( {U_{3} } \right)}}$$

**Figure 2:**
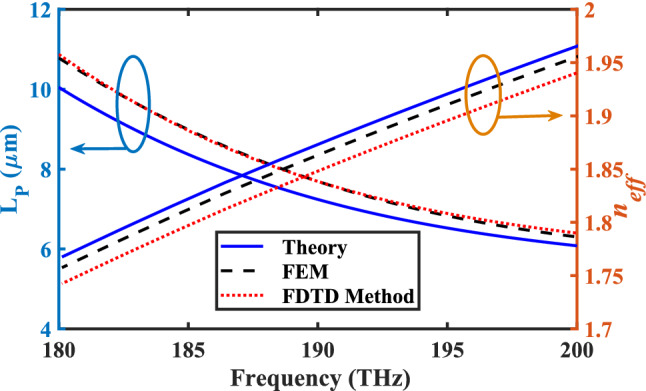
Spectra of the real part of the effective refractive index $\left( {n_{eff} } \right)$ and SPPs propagation length $\left( {L_{p} } \right)$ for the TM_01_ mode of the CHPW based on the analytical and numerical methods.

The solution of Eq. (14) provides accurate information about the guided TM_01_ mode. The Eq. (14) is solved by the genetic algorithm to find the allowed mode. This method uses a simplex search algorithm, but it finds only local solutions. To ensure that we have calculated the effective refractive index properly, we have compared the values obtained by Eq. (14) with those obtained from the FEM and FDTD simulations, as shown in Fig. 2. The effective refractive indices that are attained by the three methods are well-matched and the difference is less than 0.03% at the worst case. It is because the thickness of the InGaAs is $t_{H} = 3$ μm for numerical simulation, while it is infinite in the analytical model. It should be noted that by choosing $t_{H} = 3$ μm for numerical simulations, the infinite condition for this layer has been considered, physically.

Figure 2 also depicts the propagation length of the SPPs for both analytical and numerical methods. It is clear that based on $L_{\text{P}} = \frac{{\lambda_{0} }}{{4\pi \left| {k_{eff} } \right|}}$ formula, the propagation length (*L*_P_) is related to the imaginary part of the effective refractive index, which is directly related to the characteristics of the Au layer such as its permittivity and thickness. The specification of the Au permittivity is exactly the same for both numerical methods by choosing the best curve fitting experimental data. Therefore, the obtained results of *L*_P_ by the FEM and FDTD simulations are quite close to each other. On the other hand, the mesh size and its configuration type are totally different for FEM and FDTD numerical simulations leading to changes in the number of meshes for each layer. Extracted data such as *n*_*eff*_ and *k*_*eff*_, based on the mode analysis, are obtained from the differential equation in each zone. As a result, it is obvious that *n*_*eff*_ has bigger spread than *L*_P_ because it is related to the number of meshes in the semi-infinite thickness of InGaAs layer and other layers. Moreover, based on the nature of the non-Cartesian and regular Cartesian-grid mesh configurations, it is apparent that the obtained results of *n*_*eff*_ will not be exactly the same. In other words, as the thickness of InGaAs is greater than the other layer, the number of meshes in this layer has major effect on *n*_*eff*_. Consequently, there is a little difference between the obtained results of *n*_*eff*_ by two mentioned numerical methods.

In order to describe the properties of the CHPW, the propagation length, normalized mode area $\left( {A_{\bmod } = \frac{{A_{eff} }}{{\lambda_{0}^{2} }}} \right)$, and figure of merit (FOM) $\left( {{\text{FOM}} = \frac{{L_{{\text{P}}} }}{{\sqrt {{{A_{eff} } \mathord{\left/ {\vphantom {{A_{eff} } \pi }} \right. \kern-\nulldelimiterspace} \pi }} }}} \right)$ are studied. The effective mode area $\left( {A_{eff} } \right)$ is defined as^19^:

16a $$A_{eff} = \frac{{\iint {W(r)}d^{2} r}}{{\max \left\{ {W(r)} \right\}}}$$

16b $$W(r) = \frac{1}{2}\left\{ {{\text{Re}} \left[ {\frac{{d\left( {\varepsilon (r)\omega } \right)}}{d\omega }} \right]\left| {{\text{E}}\left( r \right)} \right|^{2} + \mu_{0} \left| {{\text{H}}\left( r \right)} \right|^{2} } \right\}$$

where ${\text{E}}\left( r \right)$ and ${\text{H}}\left( r \right)$ are the electric and magnetic fields, respectively, and $\varepsilon \left( r \right)$ is the permittivity of different regions. The obtained $A_{\bmod }$ and FOM are 0.05 and 41.02, respectively. A good design of CHPW must have simultaneously both small mode area and long propagation length, which will be a trade-off between them. Based on the obtained results, it is clear that such small mode area with long propagation length of SPPs at the wavelength of 1,550 nm confirms the ability of the proposed waveguide to break the unavoidable loss-confinement trade-off, which is desirable for integrated circuits.

**Figure 3:**
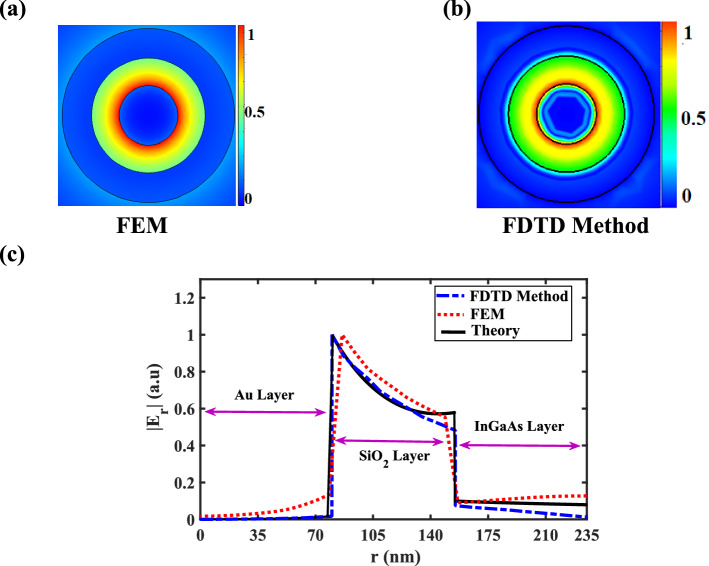
(**a**) Distribution of the normalized electric field amplitude $\left( {\left| {E_{r} } \right|} \right)$ of the TM_01_ mode at the cross-section of the CHPW based on the (**a**) FEM and (**b**) FDTD method, and (**c**) normalized amplitude of the electric field along the *r* direction when z = 0 at 193.5 THz based on the analytical and numerical methods.

The distribution of the *r*-component amplitude of the electric field $\left( {\left| {E_{r} } \right|} \right)$ at the cross-section of the CHPW is depicted in Fig. 3a,b based on the FEM and FDTD method, respectively. The profile of the electric field based on two numerical methods shows that the stimulated SPPs are centralized in the SiO_2_ layer. Moreover, as demonstrated in Fig. 3c, the numerical results of the electric field amplitude along the *r* direction are well-matched with theoretical one. A little difference between the results is related to the different mesh configurations, mesh sizes, relative errors and considered simplification to obtain the dispersion relation.

Obviously, beyond the diffraction limit, the fundamental TM_01_ mode can be confined in the thin SiO_2_ layer. Consequently, by introducing the CHPW, the electric field of the optical principle mode has been tightly limited in the low refractive index layer.

## Characteristics of the circular hybrid plasmonic waveguide-fed nano-antenna

**Figure 4:**
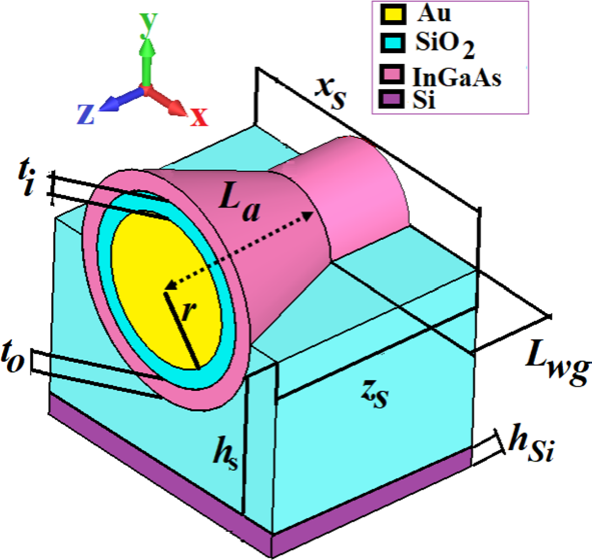
The 3D perspective view of the proposed circular hybrid plasmonic nano-antenna. The thicknesses of the Si substrate, SiO_2_ and InGaAs layers are $h_{Si} = 220\,\,{\text{nm}}$, $t_{i} = 75{\text{ nm}}$ and $t_{o} = 80{\text{ nm}}$, respectively. The radius of the gold is $r = 300\,\,{\text{nm}}$. The length of the CHPW is *L*_*wg*_ = 375 nm and $h_{s}$ and *L*_*a*_ are 830 and 575 nm, respectively. The foot-print of the proposed nano-antenna is $x_{s} \times z_{s} = 1200 \times 950\,\,{\text{nm}}^{{2}}$_._

The first step to design our proposed nano-antenna is to develop a wideband nano-antenna with low loss, low reflection, high directivity, high efficiency, and a reduced transverse size. Despite some studies to develop optical features of HPNA^20–26^, no study has been done about the CHPWFNA. The horn nano-antenna, flared to a larger opening, is so useful in RF region because they can make uniform phase front to enhance the directivity and gain^32^. Therefore, we have proposed a CHPWFNA, which is excited by the CHPW. The 3D schematic view of the proposed CHPWFNA is illustrated in Fig. 4. The values of the geometrical parameters have been chosen by parametric analysis and optimization module with the goal of maximizing the gain and directivity of the proposed nano-antenna.

It is worth noting that the thickness and material of substrate have been selected according to the technological challenges, limits and constraints to avoid complexity in the fabrication process^33^. Moreover, to compatible our proposed structure with silicon nanophotonic devices and circuits, choosing an appropriate substrate is quite important. Typically, silicon (Si) is a good choice because silicon on insulator (SOI) wafers are widely used in silicon nanophotonics. A silicon wafer is made up from a thin flat slice of large, single and defect-free crystalline silicon, which is a suitable platform as a substrate. The stability of silicon as a semiconductor material is quite high and so it is absolutely appropriate for the fabrication of integrated circuits^34^. The purity of single-crystal silicon, which is used to build wafers, is more than 99.9999999%. The necessity of such silicon purity provides remarkably technical challenges in its fabrication. A silicon crystal contains 5 × 1,022 atoms per cm^3^, which its impurity concentrations is less than one part per trillion^34^. The Czochralski method is a crystal growth technology for highly pure materials such as silicon^34,35^. In SOI technology, the thickness of Si substrate is considered typically 220 nm to decrease the parasitic capacitance within the device^36^. Consequently, the thickness of the substrate is not used as a geometrical parameter for tailoring and optimizing and it is set as 220 nm based on the commercial wafer.

To excite the proposed nano-antenna a waveguide port is used, which stimulates the CHPW connected to the radiation part of the nano-antenna. Also, the eigenmode solver is utilized to calculate the exact port mode (TM_01_ mode) within the low refractive index layer^25^.

**Figure 5:**
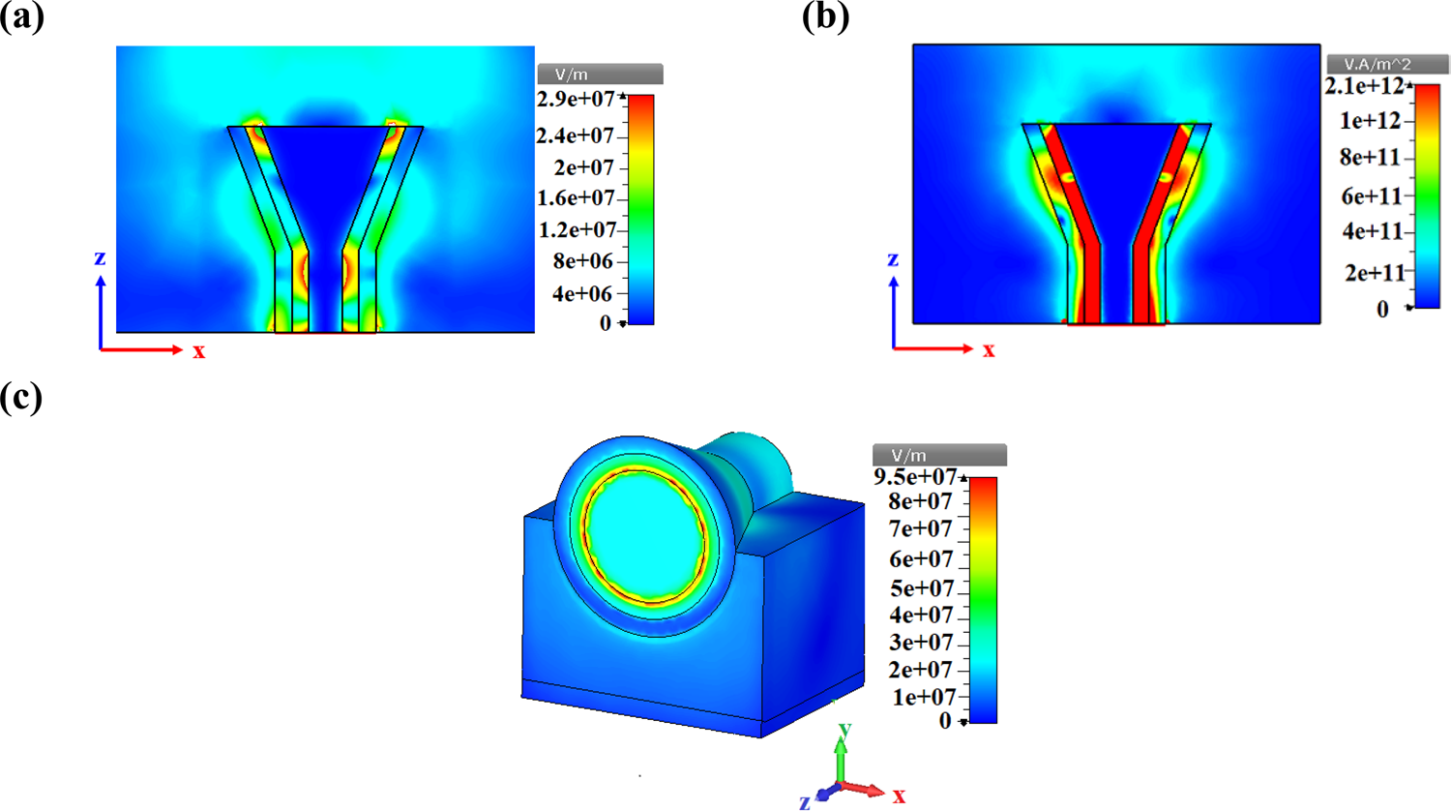
Two-dimensional (2D) view of the near-field electric field of the CHPWFNA at the wavelength of 1,550 nm (193.5 THz) at the xz plane based on the (**a**) E-field and (**b**) intensity distributions and (**c**) 3D perspective view. All structural parameters are the same as Fig. 4.

To better appreciate how the excited SPPs feed the proposed CHPWFNA, the two- and three-dimensional near-field distributions of the CHPWFNA at the wavelength of 1,550 nm (193.5 THz) should be studied. As illustrated in Fig. 5a,b, the propagated SPPs are easily concentrated at the thin SiO_2_ circular layer. Also, based on Fig. 5a, it is clear that SPPs radiate to free space at the end of the antenna to make the radiation pattern. Therefore, the plasmonic manner of the proposed antenna is realized by study the E-filed and intensity distributions at the desired wavelength. Moreover, as plotted in Fig. 5c, 3D near-field distribution confirms that TM_01_ fundamental mode is confined at the SiO_2_ layer.

To investigate the performance of the CHPWFNA, the radiation pattern, directivity and realized gain should be calculated. The realized gain (*RG*) and directivity (*D*) are calculated as^37^:

17 $$RG\left( {\theta ,\phi } \right) = 4\pi \frac{{I\left( {\theta ,\phi } \right)}}{{P_{in} }},{\text{ D}}\left( {\theta ,\phi } \right) = 4\pi \frac{{I\left( {\theta ,\phi } \right)}}{{P_{rad} }}$$

where $I\left( {\theta ,\phi } \right)$ is the angular radiated intensity, $P_{in}$ and $P_{rad}$ are the input simulated power to the CHPW and total radiated power, respectively. $P_{in}$ is calculated from the power that is delivered by the signal generator to the waveguide port. Furthermore, $P_{rad}$ is calculated by setting far-field monitors for different frequencies^25^. It means that each defined far-field monitor adds a corresponding power value to the radiated power, which should be computed by integrating the power radiated in the volume by the radiator. $I\left( {\theta ,\phi } \right)$ and radiation pattern have been obtained by performing the standard near-to-far field projections of the fields recorded for different frequencies based on the simulated electromagnetic fields on a closed box surrounding the CHPWFNA^25^.

**Figure 6:**
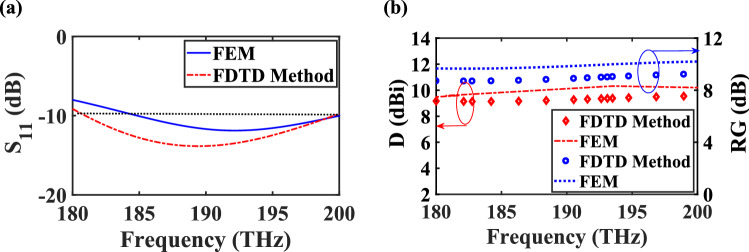
(**a**) Spectra of the reflection coefficient, (**b**) realized gain and directivity of the proposed CHPWFNA based on the FEM and FDTD method.

The reflection coefficients (S_11_) of the proposed CHPWFNA based on the FEM and FDTD simulations are depicted in Fig. 6a and show that the proposed nano-antenna work in the frequency range of 185 to 200 THz, which covers standard optical communication bands of S and C.

The study of realized gain, which includes the mismatch and mispolarization losses, reveals that the nano-antenna can effectively convert the input power to the radiated power at a desired wavelength. Also, the directivity of the CHPWFNA shows its ability to direct the far-field radiation and enhance the intensity confinement at a desired direction. Therefore, the realized gain and directivity are obtained based on the FEM and FDTD method and demonstrated in Fig. 6b. The attained realized gains (directivities) at the wavelength of 1,550 nm based on the FDTD and FEM numerical simulations are 9.03 dB (9.38 dBi) and 10.00 dB (10.32 dBi), respectively. It reveals that the FDTD results are in good agreement with ones obtained from the FEM. The difference is less than 5% at the worst case.

The total efficiency is defined as the ratio of the total radiated power to the accepted power by the CHPW from the connected transmitter, which takes into account the reflection, conduction and dielectric losses. The total efficiencies are obtained as − 0.35 dB (92.25%) and − 0.32 dB (92.89%) at the wavelength of 1,550 nm for the FDTD and FEM numerical simulations, respectively.

It is essential to study about a slightly difference between the results obtained by the FEM and FDTD method. One of the most important factors is mesh configuration. The non-Cartesian and regular Cartesian-grid meshes are used for the FEM and FDTD method, respectively. In contrast to the regular Cartesian-grid, the FEM grid is defined by the nodes on the boundary of the nano-antenna. Consequently, the FEM grid matches closely the geometry of the nano-antenna. The other factor is convergence of the numerical method, which is related to the relative error. It is computed by comparing the solutions obtained with two successive grid refinements. As the relative error is different for both numerical methods, this dissimilarity between the numerical results is obvious. Also, the computation time does not have effect on the obtained results by the numerical methods, but it should be mentioned that, in this case, utilizing FDTD procedure can greatly reduce the computation time, especially when a large number of wavelengths is of interest.

**Figure 7:**
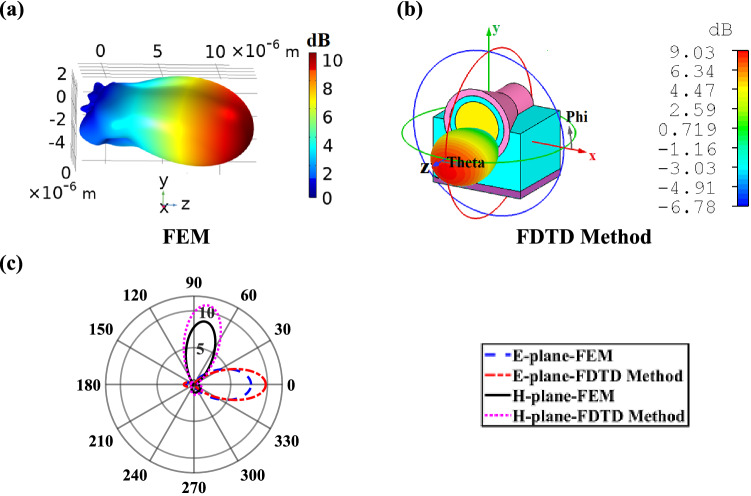
3D radiation realized gain pattern based on (**a**) FEM and (**b**) FDTD method and (**c**) E-plane and H-plane directivity patterns (in linear scale) at the wavelength of 1,550 nm based on the FEM and FDTD method. All dimensions are the same as Fig. 4.

The 3D radiation realized gain patterns of the proposed nano-antenna and E-plane and H-plane directivity patterns in linear scale are illustrated in Fig. 7. The CHPWFNA radiates a linearly polarized field with the main E-field and H-field components at the yz and xz planes, respectively. Moreover, the side lobes level is less than − 10 dB, the radiation pattern is shifted about $- 10^{ \circ }$ relative to the *z*-axis and the angular widths (3 dB) of the E-plane and H-plane are $\theta_{E}$ = 62.00° (48.80°) and $\theta_{H}$ = 50.30° (50.30°) at 1,550 nm for the FDTD method (FEM).

**Figure 8:**
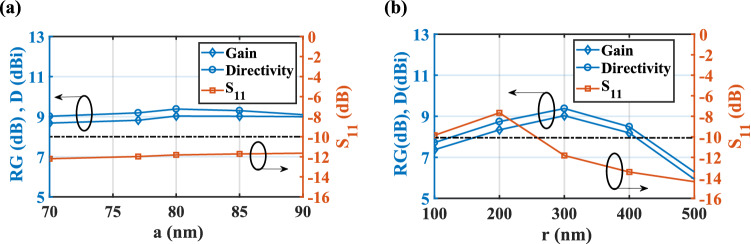
Effect of the radius of the gold layer in the (**a**) CHPW and (**b**) radiation part of the CHPWFNA on the realized gain, directivity and reflection coefficient. $t_{i} = 75\,\,{\text{nm}}$, $t_{o} = 80\,\,{\text{nm}}$, *L*_*wg*_ = 375 nm, and *L*_*a*_ = 575 nm. The dash-dot line represents S_11_ = − 10 dB.

Here, the effect of structural parameters on the nano-antenna performance has been studied. As shown in Fig. 8a, by increasing the radius of the gold nanowire in the CHPW part from 70 to 90 nm, although the reflection coefficient is less than − 10 dB, the best efficiency of 92.25% is attained for *a* = 80 nm. Also, the effect of the radius of the gold layer in the radiation part is illustrated in Fig. 8b. It is clear that for the radii of 100 and 200 nm the reflection coefficient is greater than − 10 dB. Therefore, the obtained realized gain and directivity are not reliable.

**Figure 9:**
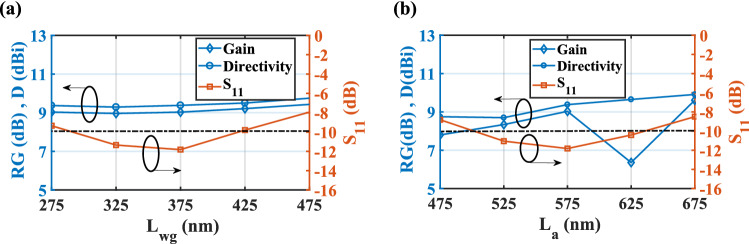
Effect of the length of the (**a**) CHPW (*L*_*wg*_) and (**b**) radiation part (*L*_*a*_) of the CHPWFNA on the realized gain, directivity and reflection coefficient. $t_{i} = 75\,\,{\text{nm}}$, $t_{o} = 80\,\,{\text{nm}}$, *a* = 80 nm, and *r* = 300 nm. The dash-dot line represents S_11_ = − 10 dB.

Based on Fig. 9a, the variation of the CHPW length (*L*_*wg*_) reveals that for the lengths of 425 and 475 nm the mismatch between the waveguide and radiation part happens and so the reflection coefficient is greater than − 10 dB. For these lengths, the realized gain and directivity have the maximum value, but they are not valid. As plotted in Fig. 9b, for the radiation part length of *L*_*a*_ = 675 nm, the obtained efficiency is maximum, but the bandwidth is decreased from 20 to 14 THz. Therefore, to have a balance between the bandwidth and efficiency, the best value of *L*_*a*_ is 575 nm.

**Figure 10:**
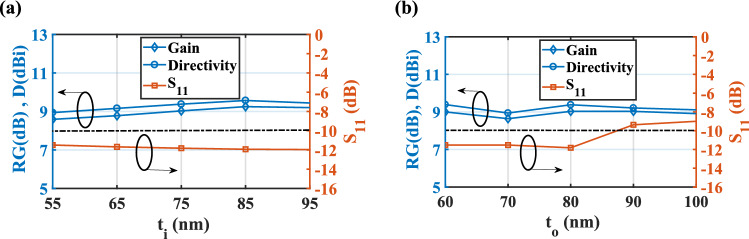
Effect of the thichness of the (**a**) SiO_2_ layer and (**b**) InGaAs layer of the CHPWFNA on the realized gain, directivity and reflection coefficient. *a* = 80 nm, and *r* = 300 nm, *L*_*wg*_ = 375 nm, and *L*_*a*_ = 575 nm. The dash-dot line represents S_11_ = − 10 dB.

Moreover, as demonstrated in Fig. 10a, by increasing the thickness of the SiO_2_ layer (*t*_*i*_) from 55 to 85 nm, both realized gain and directivity are enhanced to 9.25 dB and 9.57 dBi, respectively and then they decreased. However, for *t*_*i*_ = 75 nm, the bandwidth is maximum. Therefore, to have a reasonable relation between the gain, directivity, and bandwidth, the optimized value of *t*_*i*_ is chosen 75 nm. Also, it can be seen form Fig. 10b that the best impedance matching and maximum efficiency of 92.25% are obtained for the InGaAs thickness of *t*_*o*_ = 80 nm.

## Applications of the on-chip circular hybrid plasmonic waveguide-fed nano-antenna

To better understand the fundamental performance of the proposed CHPWFNA as a wireless point-to-point transmitting and receiving nano-antennas, the link power budget and the attenuation introduced by the free space propagation should be calculated. The performance of nano-antenna as a wireless link has been investigated by the FDTD and theoretical methods.

**Figure 11:**
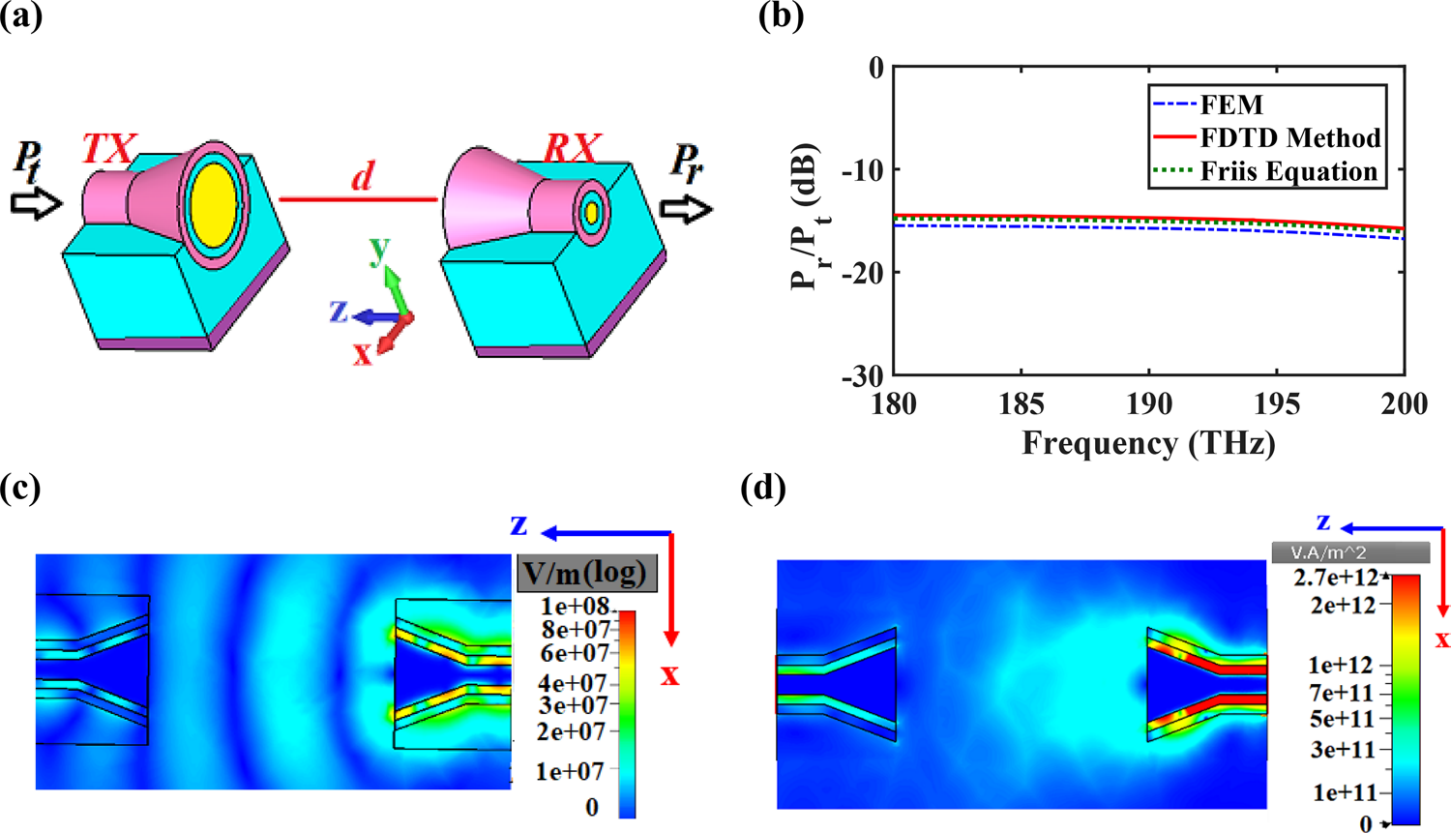
(**a**) Schematic view of a wireless link, (**b**) the ratio of the received power (*P*_*r*_) to the input power (*P*_*t*_) versus the frequency based on the mentioned numerical methods and Friis equation. (**c**) E-field and (**d**) intensity distributions between two CHPWFNAs at 1,550 nm. *d* = 5 µm and the other parameters are the same as Fig. 4.

The schematic view of an on-chip wireless link is depicted in Fig. 11a. Based on the Friis transmission equation the ratio of the received power $\left( {P_{r} } \right)$ to the transmitted power $\left( {P_{t} } \right)$ can be obtained as^38^:

18 $$\frac{{P_{r} }}{{P_{t} }} = e_{t} D_{t} e_{r} D_{r} \left| {{\text{p}}_{{\text{t}}} {\text{.p}}_{{\text{r}}} } \right|^{2} \left( {\frac{{\lambda_{0} }}{4\pi nd}} \right)^{2}$$

where $D_{t} = D_{r}$ and $e_{t} = e_{r}$ are total directivities and efficiencies of the transmitting and receiving antennas, respectively. Also, $\left| {{\text{p}}_{{\text{t}}} {\text{.p}}_{{\text{r}}} } \right|^{2}$ shows the mismatch polarization, $\left( {\frac{{\lambda_{0} }}{4\pi nd}} \right)^{2}$ is the free space attenuation, where *n* is the refractive index of the medium. The distance between the transmitter and receiver terminals (*d*) is 5 μm. As the axial ratio at the wavelength of 1,550 nm is 11 dB, the polarization of the transmitter and receiver antennas is linear. Therefore, we have considered $\left| {{\text{p}}_{{\text{t}}} {\text{.p}}_{{\text{r}}} } \right|^{2} = 1$.

The quality factors $\left( {Q = e_{t} D_{t} e_{r} D_{r} } \right)$ of the wireless link are obtained as 63.97 (18.06 dB) and 100 (20 dB) for the FDTD and FEM numerical simulations, respectively. As a result, the ratio of *P*_*r*_ to *P*_*t*_ are attained as − 14.09 and − 12.15 dB based on the Friis equation, where the directivity and efficiency results are obtained by the FDTD and FEM numerical simulations, respectively. Also, the obtained ratios of *P*_*r*_ to *P*_*t*_, shown in Fig. 11b, are in good agreement with the Friis equation results. Moreover, Fig. 11c,d demonstrates the E-field and intensity distributions between two CHPWFNAs at 1,550 nm. We can clearly observe the decay of the electric field amplitude of the propagated wave and recollecting the power in the receiving nano-antenna based on the reciprocity theory.

**Figure 12:**
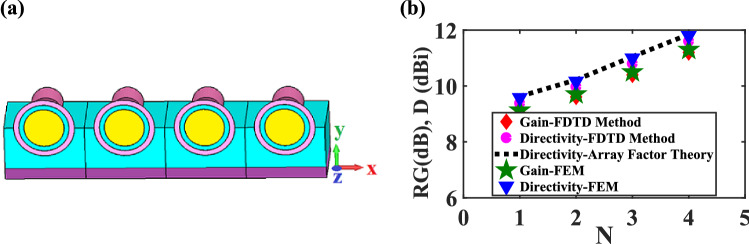
(**a**) Scheme of a single row array of four CHPWFNAs. (**b**) The realized gain and directivity for different elements numbers in a single row array based on the array factor theory, FEM and FDTD method.

Finally, the performance of the proposed nano-antenna for single row array, which is depicted in Fig. 12a, is investigated. Based on the antenna theory, it is obvious that utilizing an array of nano-antennas increases the directivity and gain. In particular, the array directivity can be calculated from the multiplication of the single CHPWFNA directivity by the array factor. Figure 12b depicts the array directivity and gain as a function of the number of antennas (N) obtained from the array factor formula, FDTD and FEM techniques.

It is essential to mention that the CHPWFNAs are excited independently in-phase, i.e. without considering the signal splitter, exciting each waveguide with its fundamental mode^26^. We can see that the FDTD and FEM results agree well with the ones obtained from the antenna theory. Also, the important consideration is the distance between the adjacent nano-antennas. The gaps between two adjacent elements are set to be $\lambda_{{_{eff} }} > 420\,\,{\text{nm}}$, where $\lambda_{{_{eff} }} > \frac{{\lambda_{c} }}{{2n_{eff} }}$ and $\lambda_{c}$ is the central wavelength of the bandwidth. Also, as the antenna foot-print is 1,200 nm along the x-axis, the best gap between two adjacent nano-antennas is 600 nm > 420 nm, which is enough to overcome the coupling effect between them.

The antenna with high gain is appropriate for energy harvesting, which can be obtained by creating an array of nano-antennas. As shown in Fig. 12, by increasing the number of nano-antenna elements, the realized gain is enhanced, which confirms the performance of the proposed nano-antenna for energy harvesting application.

**Figure 13:**
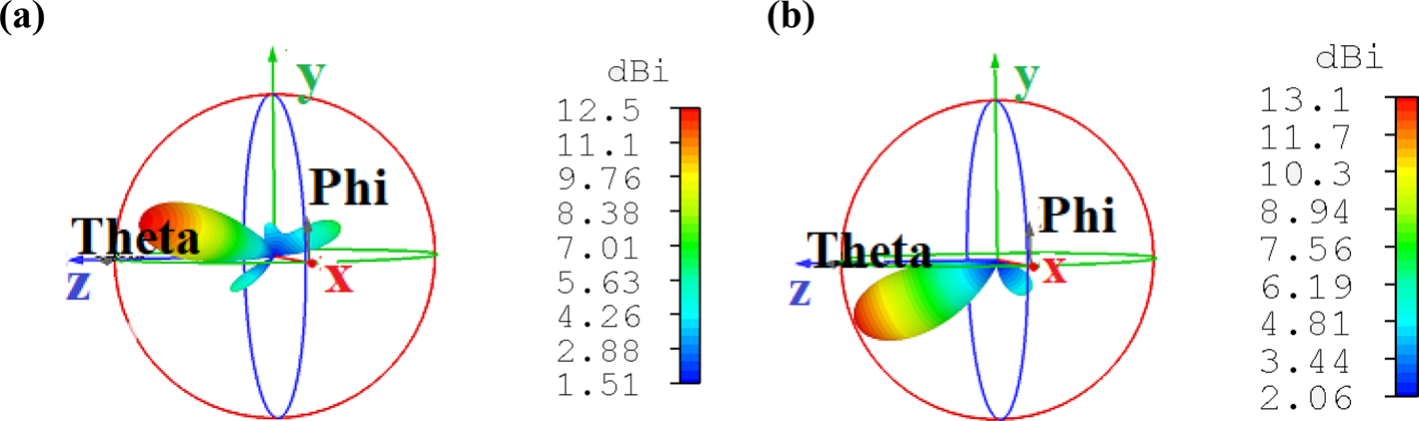
Feasibility of the proposed CHPWFNA for beam steering application. 3D directivity radiation pattern of a 3 × 3 array of the proposed nano-antenna when (**a**) $\Delta \varphi = - 90^{ \circ }$ and (**b**) $\Delta \varphi = + 90^{ \circ }$ at 1,550 nm.

Beam steering can be achieved by controlling the relative phase between the antenna elements^20^. Figure 13 shows how the direction of the pattern will be changed when the phase between a 3 × 3 array of the CHPWFNAs modifies from $\Delta \varphi = - 90^{ \circ }$ to $\Delta \varphi = + 90^{ \circ }$. Also, this figure reveals that the direction of the pattern can be steered by controlling the relative phase shift between the input optical signals in each feeding waveguide. This could be achieved, for example, by integrating highly efficient active phase shifters into each arm of the antenna array^39^.

To compare our proposed CHPWFNA and previous works, it is essential to pay attention to some points such as type of material and waveguide topology, introducing analytic approach, gain and directivity, as listed in Table 1. According to this table, it is essential to say that, for the first time, a CHPW is utilized to design a hybrid plasmonic nano-antenna. Also, two different methods are used to confirm the validity of our proposed nano-antenna performance. Moreover, compactness is one of the most important point of view to show the superiority of our structure in comparison to the plasmonic nano-antennas^40,41^. The foot-print of our nano-antenna is $1200 \times 950{\text{ nm}}^{2}$, which is more compact at least in z direction in comparison to refs.^40,41^. Also, by comparing our structure to gold nano-loop (GNL) nano-antenna^42^, the gain and directivity are increased.

**Table 1:** Specifications of our proposed on-chip CHPWFNA compared to the previous works at 1,550 nm.

References	Material	Waveguide topology	Method	Quality factor	Dispersion theory	Realized Gain (dB)/Directivity (dBi)	Foot-print (nm^2^)
^20^	SiO_2_	RHPW	FDTD	13	No	5.60/6.00	700 × 700
^21^	InP	RHPW	FDTD	60	No	6.60/7.00	800 × 1,100
^22^	SiO_2_	RHPW	FDTD	45.70	No	8.30/8.00	1,700 × 1,175
^23^	SiO_2_	RHPW	FDTD	–	No	5.00/–	1,100 × 850
^24^	SiO_2_	RHPW	FDTD	–	No	4.00/–	1,100 × 800
^25^	InP	RHPW	FDTD	94.57	Yes	10.00/10.70	1,000 × 900
^26^	SiN_x_	Coupled RHPW	FDTD	63.83	Yes	9.40/10.00	1,100 × 800
^40^	Si	PW	FDTD	–	No	9.90/11.00	3,000 × 3,300
^41^	Si	PW	FDTD	–	No	6.00/20.00	3,500 × 200
^42^	Au	GNL	FDTD	–	Yes	− 30.00/7.50	$-$
Our work	SiO_2_	Circular HPW	FDTD/FEM	63.97/100	Yes	9.03/9.38 10.00/10.32	1,200 × 950

## Fabrication process and deviation tolerance

To corroborate the possibility of the fabrication of the proposed nano-antenna, study on the deviation tolerance and fabrication method is indispensable. Although, technologically, the fabrication methods have been remarkably developed, it is not feasible to manufacture a structure with the precise dimensions of its designing. As a result, to consider the fabrication deficiencies, the far-field characteristics of the nano-antenna and its bandwidth have been calculated for up to at least 5% dimensions variations^33^. The mentioned results of Table 2 show that the the maximum obtained deviation is less than 3%, which is related to the radius of the gold (*r*).

**Table 2:** Fabrication deviation of structural parameters of the proposed nano-antenna.

Parameter (nm)	RG (dB)	D (dBi)	BW (THz)	Deviation (%)
Target	9.03	9.38	15	–
*t*_*i*_ = 75 ± 4	8.83/9.05	9.2/9.47	15	0.95–2.21
*t*_*o*_ = 80 ± 4	8.85/9.1	9.22/9.42	15	0.77–1.99
*L*_*a*_ = 575 ± 28	8.84/8.89	9.17/9.59	15	1.50–2.23
*L*_*wg*_ = 375 ± 18	9.01/9.01	9.31/9.31	15	0.22–0.74
*r* = 300 ± 15	8.76/8.86	9.42/9.15	15	0.42–2.99
*a* = 80 ± 4	8.91/9.01	9.24/9.42	15	0.42–1.49

**Figure 14:**
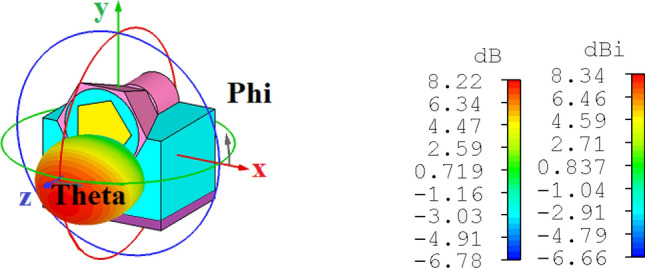
3D radiation realized gain pattern of the prosed nano-antenna with pentagonal and hexagonal cross-sections of the gold and InGaAs nanowires, respectively.

Moreover, the scanning electron microscope (SEM) observation has revealed uniform gold and InGaAs nanowires with pentagonal and hexagonal cross-sections, respectively^43–45^. Therefore, to increase the fabrication feasibility, the realized gain and directivity are calculated when we have modelled a pentagonal Au and hexagonal InGaAs nanowires for the proposed nano-antenna. As shown in Fig. 14, the obtained realized gain and directivity are 8.22 dB and 8.34 dBi, respectively. Also, the 3 dB angular width and main lobe direction for H-plane (E-plane) are $52.20^{^\circ } \,\,\left( {64.50^{ \circ } } \right)$ and $15.00^{^\circ } \,\,\left( {0^{ \circ } } \right)$, respectively.

It is essential to mention that in comparison to the circular cross section, uniform gold and InGaAs nanowires with pentagonal and hexagonal cross sections, respectively, have many effective corners. These effective corners play two crucial roles: (1) increase the continuous reflection of the confined light in the SiO_2_ layer, which leads to increasing the loss and destructive interference and decreasing the SPPs propagation when the propagated light collides with corners of the gold layer and (2) the radiated SPPs to free space from the effective corners experience a slightly different directions to propagate, which leads to the scattering of the excited SPPs^37^. As a result, the corner reflectors have a radar cross section (RCS) almost as high as the circular cross section but over a wider angle. In this case, the 3 dB angular widths of both E- and H-planes have been enhanced over 2°, which leads to decline the antenna directivity in comparison to the proposed antenna with circular cross section. Moreover, by increasing the loss due to the continuous reflections in SiO_2_ layer, the realized gain of the suggested antenna with pentagonal and hexagonal cross sections has been decreased from 9.03 dB to 8.22 dB compared to the antenna with circular cross section. Furthermore, due to increasing the scattered SPPs because of the corners, the lobe direction has been changed from − 10° to 10° relative to the z-axis.

Finally, the fabrication of high-quality gold surface with the thickness of a few nanometers is quite challenging^46^. Two parameters are commonly used to describe the characteristics of the gold layer, the gold radius (*r*) and surface roughness. Surface roughness is quantified by the deviations in the direction of the normal vector of a real surface from its ideal form. If these deviations are large, the surface is rough; if they are small, the surface is smooth^46^. In order to investigate the performance of the proposed antenna as a real device, we replace the ideal uniform gold layer with random rough surface, as shown in Fig. S1 of SI. The expression of the random surface is represented using Eq. (S8) ^46^ of SI and its related functions have been depicted in Fig. S2 of SI.

**Figure 15:**
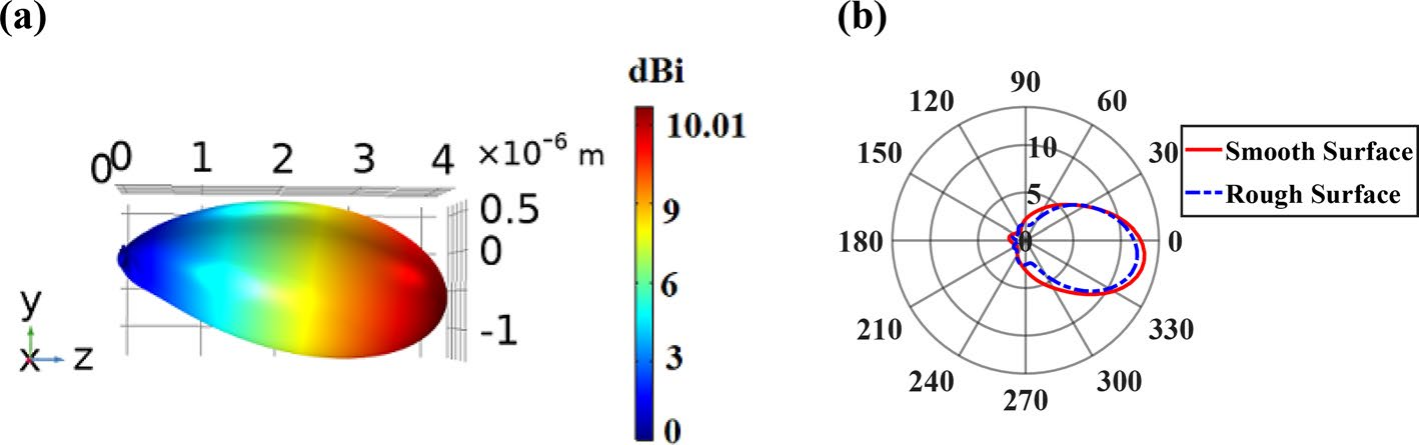
(**a**) 3D radiation directivity pattern of the proposed CHPWFNA with the random surface roughness of 0.5 nm fluctuation for the gold layer based on the FEM simulation. (**b**) 2D radiation directivity pattern of the proposed nano-antenna for both smooth and rough gold surfaces.

As illustrated in Fig. 15a, by utilizing this random surface roughness with its specific characteristic the directivity of 10.01 dBi is obtained, which is decreased about 0.31 dBi in comparison to the directivity of the antenna with smooth gold layer. By considering the surface roughness, the loss increases due to increasing the scattering of the propagated SPPs in the SiO_2_ layer. Also, it makes to attain the realized gain of 9.83 dB with − 9° main lobe direction relative to the z-axis. Moreover, to show the effect of roughness on the directivity pattern of the proposed antenna, the 2D plot of directivity for both smooth and rough gold surfaces is shown in Fig. 15b, which depicts a decrease in directivity for rough gold surface.

The CHPWFNA can be fabricated by etching a hole in the center of an InGaAs nanowire and filling the hole with the low-index shell and metal^18,44^. To realize this technique, the growth of the InGaAs and Au nanowires are carried out by molecular-beam epitaxy (MBE) and surfactant assisted chemical synthesis in acidic aqueous solution, respectively^47,48^. Also, SiO_2_ layer on the gold nanowire is produced by atomic layer deposition^49^. In order to avoid spacing between the SiO_2_ and InGaAs, soft lithographic molding and galvanic displacement deposition technique should be used^50^. Here, the SiO_2_ interlayer plays an important role to electronically passivate the surface defects of the InGaAs nanowire during atomic layer deposition^49^.

## Conclusion

In summary, an unexampled circular hybrid plasmonic waveguide-fed nano-antenna (CHPWFNA) has been proposed. An analytical approach has been used to develop the dispersion formula for design and analysis of the circular hybrid plasmonic waveguide (CHPW) characteristics as a feed of the nano-antenna. Furthermore, 3D full-wave numerical techniques of FEM and FDTD have been used to validate the theoretical approach. The proposed CHPWFNA provides the realized gain, directivity and efficiency of 9.03 dB, 9.38 dBi and 92.25% for the FDTD method, and 10.00 dB, 10.32 dBi and 92.89% for the FEM, respectively, at the standard telecommunication wavelength of 1,550 nm with the bandwidth of 20 THz. The side lobe level is less than − 10 dB, the radiation pattern is shifted about $$- 10^\circ$$ relative to the *z*-axis and the angular widths (3 dB) of the E-plane (H-plane) at 1,550 nm are $$\theta_{E} = 62.00^{ \circ }$$$$\left( {\theta_{H} = 50.30^{ \circ } } \right)$$ and $$\theta_{E} = 48.80^{ \circ }$$$$\left( {\theta_{H} = 50.30^{ \circ } } \right)$$ for the FDTD and FEM numerical techniques, respectively. As a result, both FEM and FDTD method are agree with each other and confirm the accuracy of the proposed nano-antenna performance. The suggested nano-antenna has ability to receive the electromagnetic wave from the CHPW and redirect the optical wave out of plane. Therefore, it is useful for wireless on-chip optical communications and its performance has been investigated numerically and theoretically. The obtained ratios of the received to the input powers for an on-chip wireless link, based on the Friis equation, are − 14.09 dB and − 12.15 dB at 1,550 nm for the FDTD and FEM numerical simulations, respectively. Also, the proposed point-to-point wireless link has the maximum quality factor of 63.97 (100) for FDTD method (FEM). Furthermore, by designing a 1 × 4 array of the proposed nano-antenna the maximum directivity and gain of 11.60 dBi (11.80 dBi) and 11.20 dB (11.30 dB) is obtained by the FDTD method (FEM). Finally, to consider the real condition, the effect of the pentagonal and hexagonal gold and InGaAs cross sections, respectively, and the surface roughness of the gold layer on the antenna efficiency have been investigated, comprehensively.

## Methods

The numerical analysis of the optical properties of our proposed CHPWFNA is done by 3D numerical techniques of FDTD and FEM. For the FEM simulation, the perfectly matched layer (PML) boundary condition is applied to enclose the simulation area and the whole structure is surrounded by air. The mesh size is chosen as Δx = Δy = Δz = 2 nm for the FEM simulation. For tailored accuracy requirements, the adaptive mesh refinement process is used in the FDTD simulation. The minimum number of adaptive meshing iterations is set to 15 with convergence condition of 1% maximum energy variance between adjacent iterations. To obtain the effective refractive index and excite the proposed nano-antenna by the fundamental TM_01_ mode in both 2D and 3D simulations, the optical mode solver integrated and port mode analyses are used in the FEM and FDTD solutions. Also, for far-field projections, we calculate the far-field profile on a spherical surface which is one meter far away from the simulation region. The resolution of the far-field radiation patterns is 1°. Moreover, describing the relationship between the input and output waves by S-parameters and writing these parameters in a matrix form lead to the S-matrix. In S-matrix, main diagonal elements describe the reflection at the related port. Consequently, by setting one active port as an excited port, automatically the reflection coefficient (S_11_) is calculated by both FDTD and FEM numerical simulations.

## Supplementary information

Supplementary file1
