# Supplementary material for: Theoretical analysis of a circular hybrid plasmonic waveguide to design a hybrid plasmonic nano-antenna
Source: Sci Rep. 2020 Sep 15;10:15122. doi: 10.1038/s41598-020-71863-5 (PMC7493997; doi:10.1038/s41598-020-71863-5)
Supplement: Supplementary file 1 — Supplementary file1 [file 41598_2020_71863_MOESM1_ESM.docx]

**Supplementary Information**

**Theoretical Analysis of a Circular Hybrid Plasmonic Waveguide to Design a Hybrid Plasmonic Nano-Antenna**

**Maryam Khodadadi, Najmeh Nozhat, and Seyyed Mohammad Mehdi Moshiri**

Department of Electrical Engineering, Shiraz University of Technology, Shiraz 7155713876, Iran

^*^nozhat@sutech.ac.ir

**1. Calculation method of the dispersion relation of the circular hybrid plasmonic waveguide**

To obtain the dispersion relation of the circular hybrid plasmonic waveguide, it is essential to apply the boundary conditions at the interfaces of $r=a$ and $r=b$ for satisfying the continuity of the tangential components of electric and magnetic fields across the boundaries.

At $r=a$ the boundary conditions are:

(S1-a)

(S1-b)

(S1-c)

(S1-d)

At $r=b$ the boundary conditions are:

(S2-a)

(S2-b) (S2-c)

(S2-d)

Now, we have a set of eight linear equations with eight unknown coefficients of $A_{1}, A'_{1}, A_{2}, A'_{2},A_{3}, A'_{3},B_{2},B'_{2}$ that can be organized as $\left[M\right]\left[C\right]=\left[0\right],$where $\left[M\right]$ and $\left[C\right]$ are 8×8 and 8×1 matrices, respectively and they have been extracted from equations (S1) and (S2). The determinant of matrix $\left[M\right]$ must be zero to obtain a unique dispersion relation equation. The calculation of the determinant of a 8×8 matrix is complicated. Therefore, based on equations (S1-c), (S1-d), (S2-c) and (S2-d), the unknown coefficients of $A_{1}, A'_{1}, A_{3}, A'_{3}$can be related to unknown coefficients of $A_{2}, A'_{2}, B_{2}, B'_{2}$ as follows:

$A_{1}I_{0}\left(U_{1}\right)-\left[A_{2}I_{0}\left(\frac{a}{b}U_{2}\right)+A'_{2}K_{0}\left(\frac{a}{b}U_{2}\right)\right]=0\to A_{1}=\frac{A_{2}I_{0}\left(\frac{a}{b}U_{2}\right)+A'_{2}K_{0}\left(\frac{a}{b}U_{2}\right)}{I_{0}\left(U_{1}\right)}$ (S3-a)

$A'_{1}I_{0}\left(U_{1}\right)-\left[B_{2}I_{0}\left(\frac{a}{b}U_{2}\right)+B'_{2}K_{0}\left(\frac{a}{b}U_{2}\right)\right]=0\to A'_{1}=\frac{B_{2}I_{0}\left(\frac{a}{b}U_{2}\right)+B'_{2}K_{0}\left(\frac{a}{b}U_{2}\right)}{I_{0}\left(U_{1}\right)}$ (S3-b)

$A_{3}K_{0}\left(U_{3}\right)-\text{ }\left[A_{2}I_{0}\left(U_{2}\right)+A'_{2}K_{0}\left(U_{2}\right)\right]=0\to A_{3}=\frac{A_{2}I_{0}\left(U_{2}\right)+A'_{2}K_{0}\left(U_{2}\right)}{K_{0}\left(U_{3}\right)}$ (S3-c)

$A'_{3}K_{0}\left(U_{3}\right)-\left[B_{2}I_{0}\left(U_{2}\right)+B'_{2}K_{0}\left(U_{2}\right)\right]=0\to A'_{3}=\frac{B_{2}I_{0}\left(U_{2}\right)+B'_{2}K_{0}\left(U_{2}\right)}{K_{0}\left(U_{3}\right)}$ (S3-d)

By setting equations (S3 (a-d)) into equations (S1 (a-b)) and (S2 (a-b)), we can decrease the elements number of matrix $\left[C\right]$ from eight to four, as follows:

$\left[A_{2}I_{1}\left(\frac{a}{b}U_{2}\right)+A'_{2}K_{1}\left(\frac{a}{b}U_{2}\right)\right]-\frac{U_{2}}{U_{1}}\frac{a}{b}\frac{\varepsilon_{Au}}{\varepsilon_{L}}I_{1}\left(U_{1}\right)\left[\frac{A_{2}I_{0}\left(\frac{a}{b}U_{2}\right)+A'_{2}K_{0}\left(\frac{a}{b}U_{2}\right)}{I_{0}\left(U_{1}\right)}\right]=0$ (S4-a)

$\left[B_{2}I_{1}\left(\frac{a}{b}U_{2}\right)+B'_{2}K_{1}\left(\frac{a}{b}U_{2}\right)\right]-\frac{U_{2}}{U_{1}}\frac{a}{b}I_{1}\left(U_{1}\right)\left[\frac{B_{2}I_{0}\left(\frac{a}{b}U_{2}\right)+B'_{2}K_{0}\left(\frac{a}{b}U_{2}\right)}{I_{0}\left(U_{1}\right)}\right]=0$ (S4-b)

$\left[A_{2}I_{1}\left(U_{2}\right)+A'_{2}K_{1}\left(U_{2}\right)\right]-\frac{U_{2}}{U_{3}}\frac{\varepsilon_{H}}{\varepsilon_{L}}K_{1}\left(U_{3}\right)\left[\frac{A_{2}I_{0}\left(U_{2}\right)+A'_{2}K_{0}\left(U_{2}\right)}{K_{0}\left(U_{3}\right)}\right]=0$ (S4-c)

$\left[B_{2}I_{1}\left(U_{2}\right)+B'_{2}K_{1}\left(U_{2}\right)\right]-\frac{U_{2}}{U_{3}}K_{1}\left(U_{3}\right)\left[\frac{B_{2}I_{0}\left(U_{2}\right)+B'_{2}K_{0}\left(U_{2}\right)}{K_{0}\left(U_{3}\right)}\right]=0$ (S4-d)

After considerable algebraic calculations on equations (S4 (a-d)) we have:

$\left[\frac{U_{2}}{U_{1}}\frac{a}{b}\frac{I_{1}\left(U_{1}\right)}{I_{0}\left(U_{1}\right)}I_{0}\left(U_{2}\frac{a}{b}\right)-I_{1}\left(U_{2}\frac{a}{b}\right)\right]B_{2}+\left[\frac{U_{2}}{U_{1}}\frac{a}{b}\frac{I_{1}\left(U_{1}\right)}{I_{0}\left(U_{1}\right)}K_{0}\left(U_{2}\frac{a}{b}\right)-K_{1}\left(U_{2}\frac{a}{b}\right)\right]B'_{2}=0$ (S5-a)

$\left[I_{1}\left(U_{2}\right)-\frac{U_{2}}{U_{3}}\frac{K_{1}\left(U_{3}\right)}{K_{0}\left(U_{3}\right)}I_{0}\left(U_{2}\right)\right]B_{2}+\left[K_{1}\left(U_{2}\right)-\frac{U_{2}}{U_{3}}\frac{K_{1}\left(U_{3}\right)}{K_{0}\left(U_{3}\right)}K_{0}\left(U_{2}\right)\right]B'_{2}=0$ (S5-b)

$\left[\frac{U_{2}}{U_{1}}\frac{a}{b}\frac{\varepsilon_{Au}}{\varepsilon_{L}}\frac{I_{1}\left(U_{1}\right)}{I_{0}\left(U_{1}\right)}I_{0}\left(U_{2}\frac{a}{b}\right)-I_{1}\left(U_{2}\frac{a}{b}\right)\right]A_{2}+\left[\frac{U_{2}}{U_{1}}\frac{a}{b}\frac{\varepsilon_{Au}}{\varepsilon_{L}}\frac{I_{1}\left(U_{1}\right)}{I_{0}\left(U_{1}\right)}K_{0}\left(U_{2}\frac{a}{b}\right)-K_{1}\left(U_{2}\frac{a}{b}\right)\right]A'_{2}=0$ (S5-c)

$\left[\frac{U_{3}}{U_{2}}\frac{\varepsilon_{L}}{\varepsilon_{H}}I_{1}\left(U_{2}\right)-\frac{K_{1}\left(U_{3}\right)}{K_{0}\left(U_{3}\right)}I_{0}\left(U_{2}\right)\right]A_{2}+\left[\frac{U_{3}}{U_{2}}\frac{\varepsilon_{L}}{\varepsilon_{H}}K_{1}\left(U_{2}\right)-\frac{K_{1}\left(U_{3}\right)}{K_{0}\left(U_{3}\right)}K_{0}\left(U_{2}\right)\right]A_{2}^{'}=0$ (S5-d)

The linear equations (S5(a-d)) can be written as $\left[M\right]\left[C\right]=\left[0\right]$ as follows:

$\left[M\right]\left[C\right]=\left[0\right]\to\left[\begin{matrix} a_{11}\text{ }a_{12\text{ }}0\text{ }0 \\ 0\text{ }0\text{ }a_{23}\text{ }a_{24} \\ a_{31}\text{ }a_{32}\text{ }0\text{ }0 \\ 0\text{ }0\text{ }a_{43}\text{ }a_{44} \end{matrix}\right]\text{ }\left[\begin{matrix} B_{2} \\ B'_{2} \\ A_{2} \\ A'_{2} \end{matrix}\right]=[0]$ (S6)

where

$a_{11}=\frac{U_{2}}{U_{1}}\frac{a}{b}\frac{I_{1}\left(U_{1}\right)}{I_{0}\left(U_{1}\right)}I_{0}\left(U_{2}\frac{a}{b}\right)-I_{1}\left(U_{2}\frac{a}{b}\right)$ (S7-a)

$a_{12}=\frac{U_{2}}{U_{1}}\frac{a}{b}\frac{I_{1}\left(U_{1}\right)}{I_{0}\left(U_{1}\right)}K_{0}\left(U_{2}\frac{a}{b}\right)-K_{1}\left(U_{2}\frac{a}{b}\right)$ (S7-b)

$a_{23}=\frac{U_{2}}{U_{1}}\frac{a}{b}\frac{\varepsilon_{Au}}{\varepsilon_{L}}\frac{I_{1}\left(U_{1}\right)}{I_{0}\left(U_{1}\right)}I_{0}\left(U_{2}\frac{a}{b}\right)-I_{1}\left(U_{2}\frac{a}{b}\right)$ (S7-c)

$a_{24}=\frac{U_{2}}{U_{1}}\frac{a}{b}\frac{\varepsilon_{Au}}{\varepsilon_{L}}\frac{I_{1}\left(U_{1}\right)}{I_{0}\left(U_{1}\right)}K_{0}\left(U_{2}\frac{a}{b}\right)-K_{1}\left(U_{2}\frac{a}{b}\right)$ (S7-d)

$a_{31}=I_{1}\left(U_{2}\right)-\frac{U_{2}}{U_{3}}\frac{K_{1}\left(U_{3}\right)}{K_{0}\left(U_{3}\right)}I_{0}\left(U_{2}\right)$ (S7-e)

$a_{32}=K_{1}\left(U_{2}\right)-\frac{U_{2}}{U_{3}}\frac{K_{1}\left(U_{3}\right)}{K_{0}\left(U_{3}\right)}K_{0}\left(U_{2}\right)$ (S7-f)

$a_{43}=\frac{U_{3}}{U_{2}}\frac{\varepsilon_{L}}{\varepsilon_{H}}I_{1}\left(U_{2}\right)-\frac{K_{1}\left(U_{3}\right)}{K_{0}\left(U_{3}\right)}I_{0}\left(U_{2}\right)$ (S7-g)

$a_{44}=\frac{U_{3}}{U_{2}}\frac{\varepsilon_{L}}{\varepsilon_{H}}K_{1}\left(U_{2}\right)-\frac{K_{1}\left(U_{3}\right)}{K_{0}\left(U_{3}\right)}K_{0}\left(U_{2}\right)$ (S7-h)

**2. Impact of surface roughness on the performance of the radiation pattern of proposed CHPWFNA**

In order to investigate the performance of the proposed antenna as a real device, we replace the ideal uniform gold layer with random rough surface, as shown in Fig. S1.


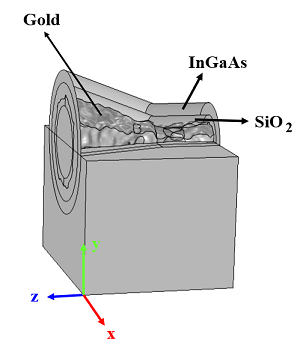


**Figure S1.** The proposed CHPWFNA with random roughness of 0.5 nm fluctuation for the gold layer.

The expression of the random surface is represented using the following formula by introducing a parametric surface, which is a 3D surface where we have used two parameters of *s_1_* and *s_2_* to define the coordinates of the surface^1^:

(S8)

where $g\left(m,n\right)$ and are a random function with Gaussian distribution and a random phase with uniform distribution, respectively, which are depicted in Fig. S2. Also, *m* and *n* are spatial frequency components, which vary from –20 to 20. Moreover, the spectral exponent *b*=1.5 indicates how quickly higher frequencies are attenuated.

| **(a)** | **(b)** |
| --- | --- |
| 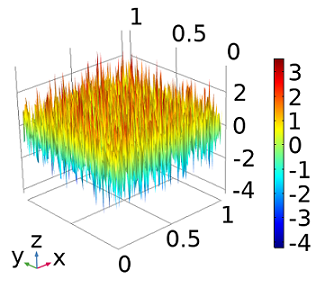 | 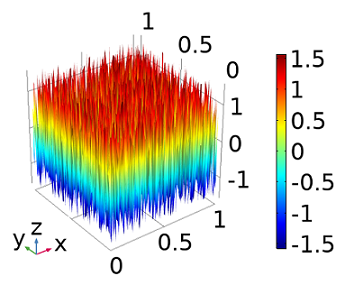 |
| **Figure S2.** The histogram of (a) a random function with Gaussian distribution and (b) a random phase with uniform distribution for applying the random roughness of 0.5 nm fluctuation for the gold layer. | |

**Reference:**

1. Xiao, Y., Qian, H., & Liu, Zh. Nonlinear metasurface based on giant optical Kerr response of gold quantum wells. *ACS photonics* **5**, 1654-1659 (2018).
